# Fabrication of Tapered Circular Depressed-Cladding Waveguides in Nd:YAG Crystal by Femtosecond-Laser Direct Inscription

**DOI:** 10.3390/mi11010010

**Published:** 2019-12-19

**Authors:** Carolina Romero, Javier García Ajates, Feng Chen, Javier R. Vázquez de Aldana

**Affiliations:** 1 Aplicaciones del Láser y Fotónica, Universidad de Salamanca, Pl. La Merced SN, 37008 Salamanca, Spain; javigarcia912@gmail.com (J.G.A.);; 2 Spanish Center for Pulsed Lasers, M5 Bldg. Science Park, Villamayor, 37185 Salamanca, Spain; 3 School of Physics, Shandong University, Jinan 250100, China; drfchen@163.com

**Keywords:** femtosecond lasers, micro-processing, photonic device, crystalline dielectrics, waveguide

## Abstract

Crystalline materials are excellent substrates for the integration of compact photonic devices benefiting from the unique optical properties of these materials. The technique of direct inscription with femtosecond lasers, as an advantage over other techniques, has opened the door to the fabrication of true three-dimensional (3D) photonic devices in almost any transparent substrate. Depressed-cladding waveguides have been demonstrated to be an excellent and versatile platform for the integration of 3D photonic circuits in crystals. Here, we present the technique that we have developed to inscribe tapered depressed-cladding waveguides with a circular section for the control of the modal behavior. As a proof of concept, we have applied the technique to fabricate structures in Nd:YAG crystal that efficiently change the modal behavior from highly multimodal to monomodal, in the visible and near infrared, with reduction factors in the waveguide radius of up to 4:1. Our results are interesting for different devices such as waveguide lasers, frequency converters or connectors between external devices with different core sizes.

## 1. Introduction

One of the most astonishing properties of femtosecond lasers is their capability to induce controlled micro-modifications in the volume of transparent dielectrics, thus allowing the integration of photonic devices with arbitrary three-dimensional (3D) geometry in almost any transparent substrate [1,2]. Glasses are the most frequently used targets to this end, provided that the fabrication of the optical waveguides on them is quite simple and straightforward [3]. Crystalline materials, however, are very attractive substrates for photonic device integration due to their excellent physical and optical properties (transparency range, non-linear behavior, spectroscopic features, etc.) that may improve the functionality of the devices but, in many cases, the inscription of waveguides cannot be done in such a direct way provided that the modification induced by the laser consists of a refractive index decrease at the focal volume. Then, other strategies must be followed, with the simplest one being the double-line waveguide [4], in which two parallel tracks are written with certain separation (typically 15–20 µm); the material stress that is produced in the central region leads to a local increase of the refractive index, thus forming a waveguide between the two tracks. Another more complex but versatile alternative is the inscription of depressed-cladding waveguides [5], which has consolidated to become an excellent platform for the integration of photonic devices in crystalline materials [6]. They consist of a tubular structure produced by multiple laser damage tracks in which the refractive index has decreased, which acts as a cladding of the waveguide, and light propagates in the central undamaged region. This type of waveguide shows interesting advantages: (i) the size and shape of the cladding can be engineered on demand so as to guide any wavelength within the whole transparency range of crystals, (ii) the modal behavior can be precisely controlled for the different spectral regions and (iii) the core region keeps the optical properties (spectroscopic, non-linear, etc.) of the bulk. For these reasons, they have been successfully employed for the fabrication of diverse functional devices, such as waveguide lasers in different spectral regions [5,7] or frequency converters by nonlinear parametric processes [8]. The main bottleneck of the technique is probably the difficulty of implementing more complex 3D photonic circuits due to the complexity of producing a continuous and smooth cladding along the whole structure. However, important steps have been taken in this direction, and efficient Y-junctions (planar and in 3D [9]), Mach–Zehnder [10] interferometers and bend waveguides [11] have been demonstrated, paving the way for the fabrication of any arbitrary 3D integrated circuit [12] in crystals based on this platform.

With the goal of increasing the functionality of the devices, other elements should be developed. A very useful one is the tapered waveguide, which is a waveguide in which the core size reduces along the propagation direction [13]. It can be used in different practical applications, such as optimizing the matching of two external waveguides with different core sizes, controlling the modal behavior along the waveguide or increasing the laser irradiance at certain sections of the waveguide. These capabilities are of great interest in active devices, as waveguide lasers or frequency converters, for instance. In glasses, tapered waveguides have been fabricated by direct femtosecond laser inscription modulating the pulse energy during the writing process [14], or by doing several laser scans [15]. However, as explained above, such techniques cannot be directly applied to many crystalline materials.

In this work, we present a technique to inscribe tapered depressed-cladding waveguides with a circular profile in crystalline materials. The 3D cladding structure consists of multiple damage tracks with a decreasing separation between them in order to fit the desired input/output radius. We have applied the technique to the fabrication of tapered structures with reduction factors up to 4:1 in Nd:YAG (neodymium doped yttrium aluminium garnet, Nd:Y_3_Al_5_O_12_) crystal, which is an excellent host material as a laser gain medium, but it can be straightforwardly applied to any other transparent crystal. Circular depressed-cladding waveguides in Nd:YAG crystal have been reported previously in literature [5,16], and perform well as integrated waveguide lasers. The tapered structures presented in this manuscript may improve the performance of these lasers, both by improving the modal behavior of the laser output, as by increasing the intensity of the pump in the tapered section.

## 2. Materials and Methods 

### 2.1. Tapered Depressed-Cladding Waveguides: The Concept and Design Parameters

The concept of a tapered depressed-cladding waveguide follows the same principles as the widespread tapered optical fibers. The direct way to control the modal behavior in tapered fiber consists of reducing the core dimensions by following a specific dependence of the fiber radius on propagation length (taper shape), which can be controlled in the fabrication procedure [17]. The length “L” of the taper determines the adiabaticity degree of the modal transformation, which is directly related to the losses induced in this section [18].

**Figure 1:**
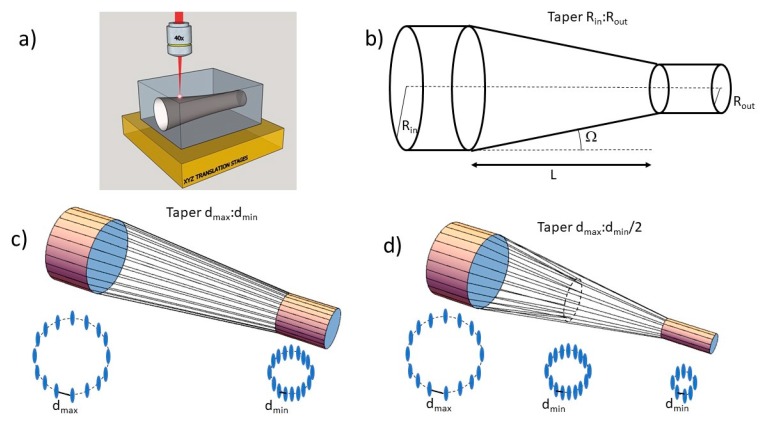
(**a**) Fabrication schematic of fs-laser inscription process. (**b**) Schematic of a tapered waveguide with *R*_in_ and *R*_out_ being the input and output cladding radius, *L* as the taper length and Ω as the taper angle. (**c**) Model for laser implementation of a tapered cladding with a reduction factor of *d*_max_:*d*_min_ by decreasing the track separation. (**d**) Model for laser implementation of a tapered cladding with a reduction factor of *d*_max_:*d*_min_/2 by decreasing the track separation and reducing the number of tracks.

In our work, the tapered structure is produced by decreasing the cladding radius from a given *R*_in_ to a *R*_out_ and by following a linear function. The length *L* of this section (see Figure 1b) defines the taper angle as:

$$\Omega =\arctan\left(\frac{R_{\mathrm{in}}-R_{\mathrm{out}}}{L}\right),$$

which is sometimes used as control parameter for tapered fiber design [13].

The cladding structures were designed and fabricated following the method presented in [10] for the fabrication of straight waveguides; the circular cladding was fitted to a polygon with a side length of “d” and with the vertices corresponding to the coordinates at which the laser damage tracks would be produced. By scanning the sample with the laser focus at these positions, straight waveguides are straightforwardly fabricated (see Figure 1a).

In order to introduce the tapered section in the waveguide, we smoothly decreased the separation between the damage tracks that constitute the cladding in such a way that there is a linear reduction of the waveguide radius along the propagation direction. This technique has two main limitations concerning the maximum reduction factor that can be achieved. On the one hand, there is a maximum separation of the damage tracks (*d*_max_) to obtain proper light confinement in the waveguide core. On the other hand, separations smaller than a certain value (*d*_min_) lead to an excess of stress accumulation in the sample and may produce a cracking of the crystal. Then, the maximum reduction factor that can be achieved with this approach is limited to *d*_max_:*d*_min_ (see Figure 1c). To increase even further the reduction factor, we have designed a powerful strategy that consists of eliminating half of the tracks once the separation between them has been decreased to the minimum accepted value (*d*_min_). In this way, the reduction factors of *d*_max_:*d*_min_/2 can be achieved (see Figure 1d), and the procedure could be applied several times to reduce further the section of the output waveguide. 

Both *d*_max_ and *d*_min_ must be experimentally determined for the optimum irradiation conditions in each crystal. This study was carried out in our case and the results are presented in Section 2.4.

### 2.2. Laser Inscription

The laser system used for inscribing the waveguides was a Ti:Sapphire based regenerative amplifier (Spitfire, Spectra Physics, Santa Clara, CA, USA), which delivers linearly polarized pulses at a repetition rate of 1 kHz. The pulses duration is 120 fs (Fourier transform limited) with a central wavelength 795 nm and a maximum energy per pulse of 1mJ. In order to reduce the amount of energy reaching the sample, we used an attenuation system formed by a halfwave plate, a linear polarizer and a set of calibrated neutral density filters.

The laser beam was focused by a 40× microscope objective (N.A. = 0.65), and the scanning of the sample was done in a three axes (XYZ) motorized stage, controlled by a Turbo Pmac controller (Delta Tau systems, Chatsworth, CA, USA). For the waveguide fabrication, the sample, an uncoated Nd:YAG (1% Nd) single crystal with a size of 10 × 10 × 3 mm^3^ and all its surfaces polished was scanned at a velocity of 500 µm/s with the beam focused at a depth of 100 µm from the top surface.

### 2.3. Optical Characterization

An optical microscope (AxioImager, Zeiss, Oberkochen, Germany) was used to inspect the inscribed structures. The near-field modal profiles at the output of the waveguides were measured with an end-fire coupling system at 633 nm (He-Ne laser) and 850 nm (laser diode). The input beam was focused at the input face of the crystal with a 10× (0.25 NA) microscope objective, and the output near-field modal profiles were recorded by imaging the waveguides with a 20× (0.40 NA) microscope objective onto a CCD camera (uEye SE, IDS, Obersulm, Germany). We also used, as a light source in this setup, a white LED in order to check incoherent and large-area un-collimated light coupling. The LED was imaged by the 10× microscope objective onto the sample surface. The propagation loss of straight waveguides was analyzed by the technique of scattered light [19], which is performed by taking an image of the waveguide from the upper part of the sample.

### 2.4. Parameters Determination

We started our study by determining the irradiation parameters for the waveguide fabrication. Firstly, we obtained the pulse energy threshold for damage focusing at a depth of 100 µm below the sample surface, which was 35 nJ. In order to get longer damage tracks, we increased the energy to 55 nJ, thus obtaining tracks with ~2 µm length. Then, we fabricated straight waveguides with *R* = 15 µm and with different values of track separation d in order to obtain the values of *d*_max_ and *d*_min_. From the analysis of the waveguides, we found that the maximum track separation for confinement at 633 nm is *d*_max_ = 4 µm; larger values lead to a very poor or negligible light confinement. On the other hand, the minimum value for which the tracks appear properly inscribed and cracking is not produced was determined to be *d*_min_ = 2 µm. Therefore, the maximum reduction factor that can be achieved directly by the technique presented in Figure 1c is 2:1, and 4:1 in the case of doing a reduction of the number of tracks is shown in Figure 1d.

**Figure 2:**
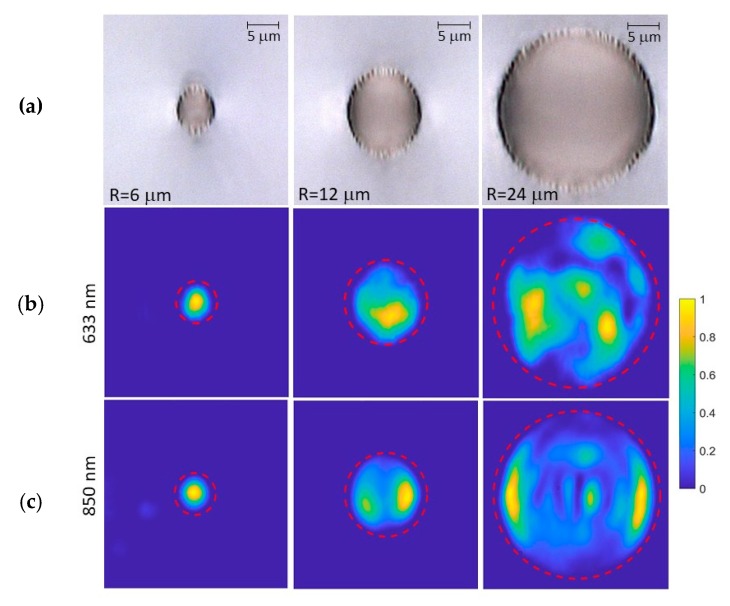
Microscopic picture taken in transmission mode of the straight waveguides fabricated with a radius of 6, 12 and 24 µm (**a**). (**b**,**c**) correspond to the modal profiles (vertical polarization) of the waveguides at 633 and 850 nm, respectively.

The next step was the determination of the optimum input/output radius for the tapers. In our design, our goal would be the transformation of a multimodal waveguide onto a monomodal waveguide, and therefore, we started establishing an optimum radius for monomodal behavior, which was found to be *R* = 6 µm; for this value, the waveguide exhibits single mode behavior at both 633 nm and 850 nm (see Figure 2, first column). Then, we fabricated straight waveguides with a radius *R* = 12 µm and *R* = 24 µm, which will be the input sizes for the 2:1 and 4:1 tapers respectively, in order to check the modal behavior. The waveguide sections and intensity profiles at output are shown in Figure 2 (central and right columns). As it can be seen, the modal profiles are complex, clearly multimode and they are dependent on the specific injection point (slight changes in the focalization point lead to different modal structures). It was not possible to excite only the fundamental mode, probably due to defects of the cladding that induce energy transfer to high order modes.

Propagation loss of the *R* = 6 µm waveguide was determined to be 1.7 dB/cm at 633 nm and 5.1 dB/cm at 850 nm. The large loss value measured for the infrared light was due to the very small core radius of the structure; a slightly larger one, at *R* = 9 µm, led to propagation loss below 3 dB/cm. However, we have decided to keep the *R* = 6 µm size in order to ensure the monomodal behavior in the visible as in the near infrared. On the other hand, the technique of scattered light method could not be applied to the *R* = 12 µm and *R* = 24 µm waveguides as it was not possible to excite only the fundamental mode, and beating between modes lead to wrong measurements.

## 3. Results and Discussion

**Figure 3:**
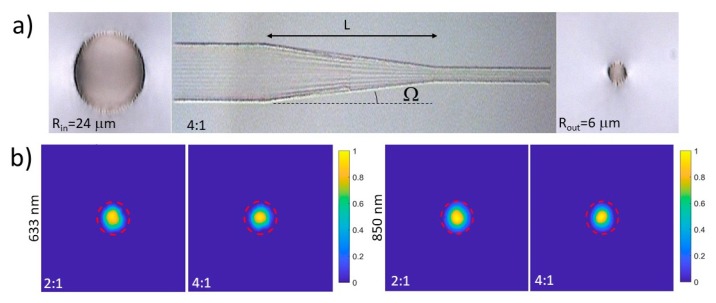
(**a**) Microscopic picture taken in transmission mode of a 4:1 tapered test structure fabricated with an input radius of 24 and an output of 6 µm (the taper length L has been shortened in order to better appreciate the details). (**b**) Modal profiles at the output of the 2:1 and 4:1 tapered waveguides at 633 nm (left) and 850 nm (right), respectively.

The 2:1 and 4:1 tapered waveguides with the selected parameters were successfully implemented in the Nd:YAG sample. In Figure 3a, it can be seen that a fabrication test of a 4:1 structure with a reduced taper length (*L*) can be used for better appreciation of the details. The plane at which half of the damage tracks end to even further reduce the radius can be seen in the central part of the picture. This plane is the most critical point due to the possibility of the crystal becoming cracked.

The final structures were fabricated with different taper lengths, from *L* = 4 mm to *L* = 6 mm. In all the cases, we found that the better performance of the structure in terms of transmitted power corresponded to the case of *L* = 6 mm. We could not test even longer values of L because we decided to keep at least 2 mm of straight waveguide at the input and output sides, and the crystal length is limited to 10 mm. For simplicity, in this work, we only show the results corresponding to the case of *L* = 6 mm.

Modal profiles of 2:1 and 4:1 tapered waveguides are shown in Figure 3b, for both 633 and 850 nm. For these cases, the taper angle was Ω = 1 mrad (2:1) and Ω = 3 mrad (4:1). It is clear from the pictures that the single mode behavior of the *R* = 6 µm straight waveguides (Figure 2) was perfectly preserved in the tapered structures.

In order to evaluate the efficiency of the modal formatting in the tapered waveguides, we have done a comparative study of the output power for both straight and tapered waveguides at the different input laser conditions, which is summarized in Table 1. Measurements have been normalized to the corresponding maximum output power. Several conclusions can be extracted from the data. Firstly, in all the cases, the maximum transmitted power is obtained for the *R* = 24 µm waveguide, followed by the *R* = 12 µm one. This is due to the larger numerical aperture of the larger radius cores that improves the coupling with the external source. Secondly, 2:1 tapered waveguides operating in the visible increase the output power in comparison with straight *R* = 6 µm waveguides. This increase can be understood as an efficient modal transition from the *R* = 12 µm to the *R* = 6 µm. Thirdly, the 4:1 tapered waveguide, although it is very efficient in generating a single mode output, it does not produce a significant increase in the output power compared to the straight waveguide. We think that in this case, a larger taper length L would be required in order to produce a quasi-adiabatic transformation of the excited input modes into the final single mode. Finally, when infrared light was used as an input in the 2:1 and 4:1 structures, the output power was generally very low, which is probably due to the non-optimized output radius for this wavelength, as explained in Section 2.4. 

**Figure 4:**
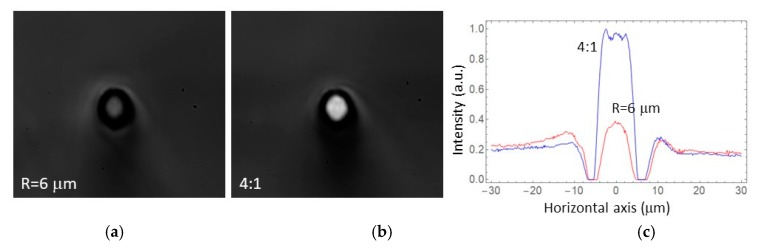
Modal profiles of (**a**) the *R* = 6 µm straight waveguide and (**b**) 4:1 tapered waveguide, illuminated with white light from an LED as an input. The plot on the right, (**c**), shows the intensity profiles (cuts) along the horizontal axis.

The behavior of the tapered waveguides with diffuse white light from an LED source was also investigated. In Figure 4, we present the results of white light coupling in the straight *R* = 6 µm waveguide and in the 4:1 structure under the same illumination conditions. In the case of the *R* = 6 µm waveguide, the intensity of coupled light was only 75% above the background level. However, in the 4:1 taper, the coupled light intensity was 5 times larger than the background level, demonstrating an effect of efficient light concentration [13].

## 4. Conclusions

The reported technique for the fabrication of circular depressed-cladding tapered waveguides in crystalline materials has been demonstrated to be versatile and robust, allowing reduction factors of the waveguide radius up to 4:1. We have successfully fabricated integrated devices that transform highly multimodal into single mode waveguides, both in the visible as near infrared. In the case of 2:1 tapers, the output power may double or triple the value of a straight waveguide with the same radius as the output, suggesting a situation of quasi-adiabaticity for the selected parameters. The 4:1 taper does not provide a power increase, probably due to a very large taper angle. Light coupling from a diffuse white-light source (white LED) was also tested, demonstrating an effect of light concentration. In summary, in our work, we have shown the design and the potential of the technique that can be applied to different devices requiring modal formatting or light concentration, but each particular one would require a specific design in order to optimize the performance.

## Figures and Tables

**Table 1:** Integrated output intensity for the different waveguides and input sources. Measurements for the same source have been normalized to the maximum value. *V* and *H* stand for vertically and horizontally polarized light, respectively (Tap. stands for Tapered).

Input Light	*R* = 24 µm	*R* = 12 µm	*R* = 6 µm	Tap. 2:1	Tap. 4:1
633 nm (*V*)	1	0.75	0.11	0.26	0.12
633 nm (*H*)	1	0.80	0.12	0.32	0.13
850 nm (*V*)	1	0.31	0.06	0.06	0.04
850 nm (*H*)	1	0.76	0.23	0.14	0.04
